# The Body Multiple: Conceptualizing the Body to Explain Functional Somatic Symptoms

**DOI:** 10.1192/bjo.2024.241

**Published:** 2024-08-01

**Authors:** Chloe Saunders, Chris Burton, Heidi Frølund Pedersen, Charlotte Rask, Lisbeth Frostholm

**Affiliations:** 1 Department for Functional Disorders and Psychosomatic Medicine, Aarhus University Hospital, Aarhus, Denmark; 2 Aarhus University, Aarhus, Denmark; 3 Academic Unit of Primary Care, University of Sheffield, Sheffield, United Kingdom; 4 Department for Child and Adolescent Psychiatry, Aarhus University Hospital Psychiatry, Aarhus, Denmark

## Abstract

**Aims:**

Functional somatic symptoms (FSS) is an umbrella term for symptoms inadequately explained by structural disease or damage. FSS show complex causality, and fall between the gaps in mainstream medical epistemology and/or mind-body dualism. Lack of explanations for FSS exacerbates uncertainty, anxiety and stigma for patients, and contributes to fragmented care and inappropriate management. We aimed to develop an open access health-educational resource that provides an acceptable, relevant, and usable explanatory model of FSS to internet users.

**Methods:**

We carried out a participatory design study to develop the website bodysymptoms.org. Explanatory concepts were developed through iterative stages of dialogue between individuals with lived experience of multi-system FSS (n = 7), researchers, healthcare professionals and designers/developers. Initial explanatory components were collected from currently existing patient education about FSS, a review of the literature, and participants’ illness narratives. Principles were developed to filter, and organize these explanatory components into a coherent model. The model was translated into 5 European languages and through iterative rounds of feedback incorporated a diverse range of perspectives.

**Results:**

We describe the explanatory model that developed through the bodysymptoms project, and considerations that arose through the dialogic process. The model is based on the body as a complex adaptive system with causal interactions operating across bio-psycho-socio-ecological levels. Mechanistic processes that can maintain persistent symptoms were chosen as the main nodes (or topics) of the model, and minor topics were structured to demonstrate interactions between mechanisms. Considerations that arose during the process included coherence across philosophic, scientific and clinical levels of explanation; a therapeutic model of agency, within which explanations empowered without blame; the need to introduce notions of biological time, like body rhythms and body memory; and the role of multi-media, embodied metaphors and lived experience narratives in communication of the explanatory model. Personalisation of the model was achieved through embedding the structure of the model into the graphical and navigational structure of the website, which allows website visitors to explore the model in a non-linear manner, tailored by relevance, acceptability, and prefered level of information.

**Conclusion:**

We present results from a research in action study to develop a novel resource for understanding functional somatic symptoms. bodysymptoms.org is based on the model of the body as a complex system that adapts in personal ways. To explain FSS there is a need for new ways to understand the body and how we become unwell. Bringing diverse perspectives into dialogue generated new forms of knowledge and allowed the power of scholarship to be harnessed for immediate shared value.

